# Structural basis and functional analysis of NMDA receptor regulation by calmodulin

**DOI:** 10.1016/j.jbc.2026.111131

**Published:** 2026-01-07

**Authors:** Aritra Bej, M. Quincy Erickson-Oberg, Aparna Nigam, Isaac Yu, Johannes W. Hell, Jon W. Johnson, James B. Ames

**Affiliations:** 1 Department of Chemistry, University of California, Davis, California; 2 Department of Pharmacology, University of California, Davis, California; 3 Department of Neuroscience and Center for Neuroscience, University of Pittsburgh, Pittsburgh, Pennsylvania

**Keywords:** calmodulin, CDD, NMDA receptor, NMR, whole-cell recording

## Abstract

The synaptic plasticity mechanisms that are thought to underlie learning and memory require Ca^2+^ influx mediated by *N*-methyl-D-aspartate receptors composed of glycine-binding GluN1 and glutamate-binding GluN2 subunits. Calmodulin (CaM) binding to the cytosolic regions in both GluN1 (residues 841–865, called GluN1-C0) and GluN2A (residues 1004–1023, called GluN2A-C0) may be important for Ca^2+^-dependent channel desensitization (CDD). Here, we report NMR, ITC and electrophysiological experiments to probe the structure and functional role of Ca^2+^-bound CaM (Ca^2+^-CaM) binding to both GluN1 and GluN2A subunits. Our ITC studies show that the GluN1-C0 peptide binds to both the N-lobe and C-lobe of Ca^2+^-CaM, whereas the GluN2A-C0 peptide binds to only the Ca^2+^-CaM C-lobe. Our NMR analysis reveals GluN2A residues (W1014 and V1018) interact with exposed hydrophobic residues in the Ca^2+^-CaM C-lobe. The NMR structure of Ca^2+^-CaM bound to the GluN1-C0 peptide indicates the two CaM lobes bind to opposite sides of the GluN1-C0 helix (C-lobe contacts M848, F852, A853 and N-lobe contacts A854, V855, W858). The GluN1 mutant F852E and the GluN2A mutant W1014E both perturbed CaM binding in ITC studies, and also diminished electrophysiologically-measured CDD, suggesting CaM interaction with these residues contributes to CDD. We propose a structural mechanism of CDD wherein channel desensitization is caused by the binding of four CaM per *N*-methyl-D-aspartate receptor subunit tetramer.

*N*-methyl-D-aspartate receptors (NMDARs) are cation-selective, Ca^2+^-permeable ionotropic glutamate receptors often localized at the postsynaptic membrane in the brain. They have critical roles in learning, memory, neural development, and synaptic plasticity (1, 2, 3, 4). Functional NMDARs are heterotetrameric channels composed of two glycine-binding GluN1 subunits and two subunits that can be any combination of glutamate-binding GluN2 (A–D) or glycine-binding GluN3 (A–B) subunits. The four GluN2 subtypes are differentially expressed in nervous tissue (5, 6). The NMDAR subunits contain a modular domain architecture (Fig. 1, *A* and *C*, and *E*) including extracellular amino-terminal domain and ligand binding domain (LBD), a central transmembrane domain, and an intracellular carboxy-terminal domain (CTD) (7, 8). Activation of NMDARs upon binding of glutamate (Glu) and co-agonist glycine opens the channels and causes an increase in intracellular Ca^2+^ concentration, thus initiating a wide range of Ca^2+^-dependent downstream signaling pathways.

**Figure 1:**
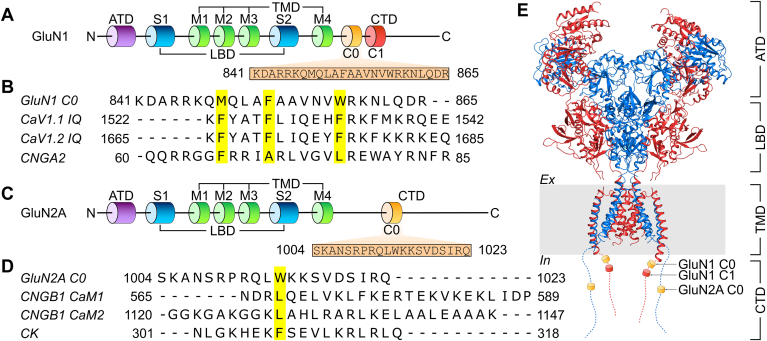
**Structural overview of NMDARs.***A*, domain architecture of GluN1. Amino acid sequence of the CaM binding site in GluN1-C0 is indicated in the *box*. *B*, multiple sequence alignment of human GluN1-C0 with the IQ motif of human Ca_V_1.1 and Ca_V_1.2, and the CaM binding site in the human olfactory cyclic-nucleotide gated channel, CNGA2. *C*, domain architecture of GluN2A. Amino acid sequence of the CaM binding site in GluN2A-C0 is indicated in the *box*. *D*, multiple sequence alignment of GluN2A-C0 with the CaM binding sites in the retinal cyclic-nucleotide gated channel (CNGB1 CaM1 and CaM2) and creatine kinase. *E*, cryo-EM structure of the *N*-methyl-D-aspartate receptor (PDB ID: 6IRA) (22). GluN1 and GluN2A subunits are colored *red* and *blue*, respectively. Functional domains are labeled as amino-terminal domain, ligand binding domain, transmembrane domain, and carboxy-terminal domain. The carboxy-terminal domain was truncated and not determined in the cryo-EM structures. Intracellular CaM binding sites (GluN1 C0, GluN1 C1, and GluN2A C0) are labeled and indicated as *cylinders*. Conserved hydrophobic residues that structurally contact CaM are shaded *yellow* in the sequence alignment.

Excessive Ca^2+^ influx through NMDARs can be cytotoxic. NMDARs are negatively regulated by intracellular Ca^2+^ elevation through a process known as either Ca^2+^-dependent inactivation or as Ca^2+^-dependent desensitization (CDD, the term that will be used here) (9, 10, 11, 12, 13). CDD of NMDARs requires direct binding of calmodulin (CaM) to the intracellular region of GluN1 (14, 15). Previous studies reveal two intracellular CaM binding sites in GluN1: residues 841 to 865 (called GluN1-C0, Fig. 1, *A* and *B*) (14) and residues 875 to 898 (called GluN1-C1, Fig. 1*A*) (16). CaM binding to GluN1-C0 was demonstrated previously to be essential for CDD (12, 14) and was suggested to promote dimerization of GluN1 (17). By contrast, the GluN1-C1 site likely does not contribute to CDD (14), but rather it controls GluN1 trafficking to the cytoskeleton (16, 18). A single CaM binding site is located in GluN2A (residues 1004–1023, called GluN2A-C0, Fig. 1, *C* and *D*) (19). Structures of NMDARs have been solved by X-ray crystallography (7, 8) and cryo-EM (20, 21, 22, 23, 24, 25). Although the structures show detailed inter-subunit interactions between the amino-terminal domain and LBD, and between the LBD and transmembrane domain, the structure of the CTD is not defined because the CTD was truncated in these studies (Fig. 1*E*). A crystal structure of CaM bound to the GluN1-C1 has been reported (16), but the structures of CaM bound to GluN1-C0 and GluN2A-C0 are still unknown. Defects in the regulation of NMDAR function are associated with a spectrum of neurological diseases and neuropsychiatric disorders (26, 27, 28, 29, 30). Elucidating NMDAR interactions with CaM may help improve our understanding of Ca^2+^-dependent channel regulation and provide a basis for treating neuronal diseases.

In this study, we present NMR-derived structures of Ca^2+^-bound CaM (called Ca^2+^-CaM) bound to GluN1-C0 and GluN2A-C0 peptides. The relevance of these structural interactions for CDD was confirmed by mutagenesis, ITC and electrophysiological measurements. Our studies are not able to detect the binding of Ca^2+^-free CaM (hereafter called apo-CaM) to either GluN1-C0 or GluN2A-C0. However, Ca^2+^-CaM binds to both GluN1-C0 and GluN2A-C0 in the nanomolar range. Our NMR structural analysis reveals both Ca^2+^-CaM lobes bind to opposite sides of the GluN1-C0 helix in contrast to GluN2A-C0, which binds only to the Ca^2+^-CaM C-lobe. On the basis of our results and previous cryo-EM structures of NMDARs, we propose a structural model of the full-length channel tetramer bound to 4 Ca^2+^-CaMs that we suggest may be essential for CDD.

## Results

### CaM binding to GluN1-C0 and GluN2A-C0 peptides

**Figure 2:**
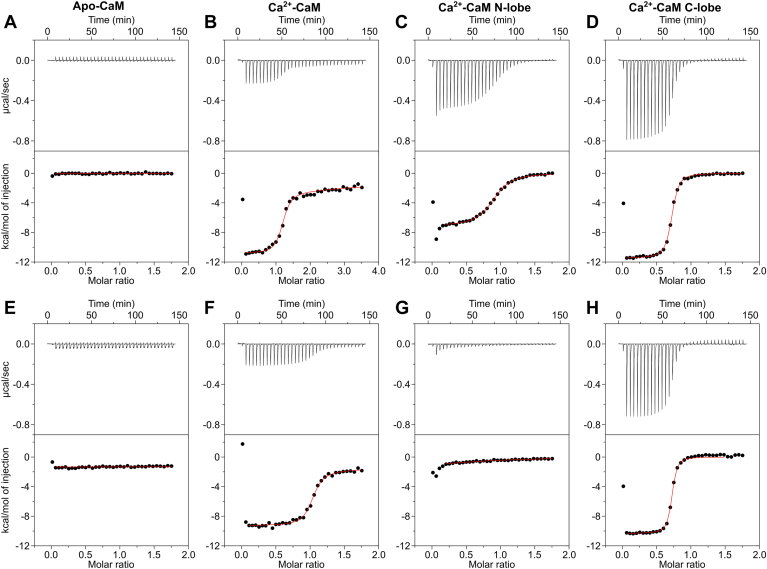
**Binding of CaM and CaM lobes to NMDAR peptides.***A*–*D*, ITC isotherms of GluN1-C0 titrated with (*A*) apo-CaM (titrand), (*B*) Ca^2+^-CaM (titrand), (*C*) Ca^2+^-CaM N-lobe (injectant), and (*D*) Ca^2+^-CaM C-lobe (injectant). *E*–*H*, ITC isotherms of GluN2A titrated with (*E*) apo-CaM (titrand), (*F*) Ca^2+^-CaM (titrand), *G*, Ca^2+^-CaM N-lobe (injectant), and (*H*) Ca^2+^-CaM C-lobe (injectant). Each isotherm was fit to a one-site model and binding parameters (Δ*H* and *K*_D_) are given in Table 1.

The affinity and stoichiometry of GluN1-C0 (or GluN2A-C0) binding to full-length CaM or to the individual N-lobe and C-lobe were determined using ITC (Fig. 2). No detectable heat signal was observed when apo-CaM was titrated with GluN1-C0 peptide (Fig. 2*A*), suggesting a lack of binding. However, previous reports suggest apo-CaM binds to GluN1-C0 in the micromolar range (12, 13). Therefore, the undetectable heat signal for apo-CaM binding to GluN1-C0 might indicate a zero change in enthalpy (ΔH = 0) rather than zero binding. The ITC isotherm of GluN1-C0 peptide binding to Ca^2+^-CaM revealed a 1:1 stoichiometry (N = 1.2 ± 0.2) and apparent dissociation constant (*K*_D_) equal to 0.06 ± 0.01 μM (Table 1 and Fig. 2*B*). GluN1-C0 peptide binds to the Ca^2+^-CaM N-lobe (residues 1–79) with a 1:1 stoichiometry (N = 0.9 ± 0.3) and *K*_D_ = 0.58 ± 0.03 μM (Table 1 and Fig. 2*C*). GluN1-C0 binds to the Ca^2+^-CaM C-lobe (residues 80–149) with a 1:1 stoichiometry (N = 0.70 ± 0.30) and *K*_D_ = 0.10 ± 0.01 μM (Table 1 and Fig. 2*D*).

**Table 1:** ITC parameters for CaM and CaM lobes binding to *N*-methyl-D-aspartate receptor peptides

Injectant/Titrand	*K* × 10^5^ (M^−1^)	*K*_D_ (μM)	*N*	Δ*H* (kcal/mol)
GluN1-C0/Apo-CaM^a^	-	ND	-	-
GluN1-C0/Ca^2+^-CaM^b^	176 ± 28	0.06 ± 0.01	1.2 ± 0.2	−10.8 ± 0.1
Ca^2+^-CaM N-lobe/GluN1-C0^b^	17 ± 1	0.58 ± 0.03	0.9 ± 0.3	−7.1 ± 0.1
Ca^2+^-CaM C-lobe/GluN1-C0^b^	103 ± 6	0.10 ± 0.01	0.7 ± 0.3	−11.5 ± 0.1
GluN2A-C0/Apo-CaM^a^	-	ND	-	-
GluN2A-C0/Ca^2+^-CaM^b^	197 ± 23	0.05 ± 0.01	1.0 ± 0.1	−9.3 ± 0.1
Ca^2+^-CaM N-lobe/GluN2A-C0^a^	-	ND	-	-
Ca^2+^-CaM C-lobe/GluN2A-C0^b^	215 ± 25	0.05 ± 0.01	0.7 ± 0.3	−10.3 ± 0.1
GluN1-C0^F852E^/Ca^2+^-CaM^b^	36 ± 4	0.28 ± 0.03	1.8 ± 0.3	−16.9 ± 0.2
Ca^2+^-CaM N-lobe/GluN1-C0^F852E^^b^	25 ± 3	0.40 ± 0.05	0.8 ± 0.3	−16.1 ± 0.3
Ca^2+^-CaM C-lobe/GluN1-C0^F852E^^b^	119 ± 7	0.08 ± 0.01	0.8 ± 0.3	−17.3 ± 0.1
GluN1-C0^W858E^/Ca^2+^-CaM^a^	1.8 ± 0.3	5.5 ± 0.8	ND	−1.2 ± 0.2
Ca^2+^-CaM N-lobe/GluN1-C0^W858E^^a^	-	ND	-	-
Ca^2+^-CaM C-lobe/GluN1-C0^W858E^^b^	1.1 ± 0.3	9 ± 2	0.7 ± 0.3	−2.7 ± 0.3
GluN2A-C0^W1014E^/Ca^2+^-CaM^a^	-	ND	-	-
Ca^2+^-CaM C-lobe/GluN2A-C0^W1014E^^a^	-	ND	-	-

a The titration curve cannot be accurately analyzed.

b Exothermic heat signals were fit to a one-site model. “ND” stands for not detected by ITC. “*N*” represents binding stoichiometry that is calculated as the molar ratio ([injectant]/[titrand]) at the inflection point in the ITC isotherm. ΔH represents the total heat (integrated from the isotherm) divided by the number of moles of titrand.

Similar ITC experiments characterized CaM binding to the GluN2A peptide. Apo-CaM lacked detectable binding to GluN2A-C0 (Fig. 2*E*), in contrast to Ca^2+^-CaM that exhibited nanomolar binding (Table 1 and Fig. 2*F*), consistent with previous observations of Ca^2+^-CaM binding to GluN2A-C0 (19). The ITC isotherm for GluN2A-C0 peptide binding to Ca^2+^-CaM was fit to a one-site model (N = 1.02 ± 0.1) with *K*_D_ equal to 0.05 ± 0.01 μM (Table 1 and Fig. 2*F*). The ITC isotherm for GluN2A-C0 peptide binding to the Ca^2+^-CaM C-lobe has a *K*_D_ of 0.05 ± 0.01 μM (Table 1 and Fig. 2*H*), which is identical to that of full-length CaM. The binding of GluN2A-C0 peptide to the Ca^2+^-CaM N-lobe produced weak ITC heat signals that could not be accurately analyzed (Fig. 2*G*). In summary, the ITC results reveal that GluN2A-C0 peptide has nanomolar binding to the Ca^2+^-CaM C-lobe and does not bind to the isolated N-lobe.

### NMR-derived structure of Ca^2+^-CaM bound to GluN1-C0 peptide

**Figure 3:**
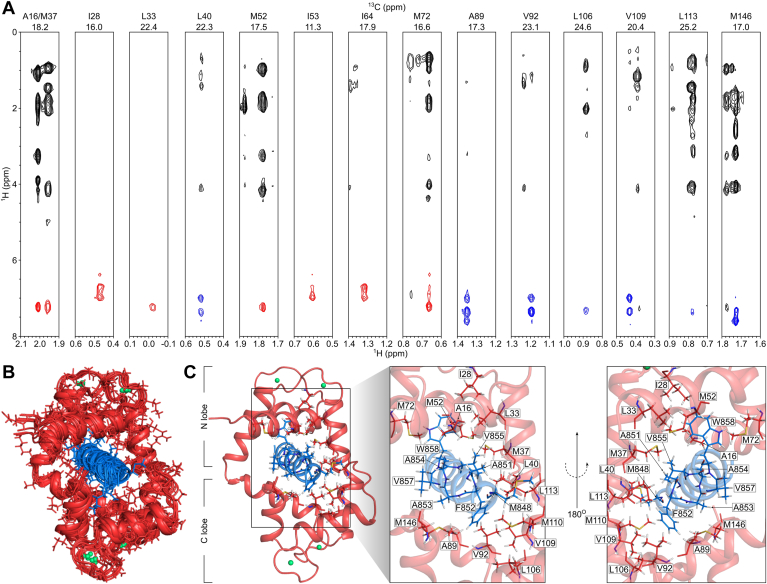
**Intermolecular interactions between Ca^2+^-CaM and GluN1-C0 peptide.***A*, ^13^C-edited (F2) and ^13^C-filtered (F1) nuclear overhauser effect spectroscopy strip plots of ^13^C, ^15^N-labeled Ca^2+^-CaM bound to unlabeled GluN1-C0 peptide. Aromatic ring protons assigned to F852 and W858 are colored *blue* and *red*, respectively. *B*, overview of the 10 best structures of Ca^2+^-CaM (in *red*) bound to GluN1-C0 (in *blue*) based on HADDOCK score. Ca^2+^ ions are colored in *green* spheres. Residues in the lowest HADDOCK score structure were highlighted in lines. *C*, ribbon representation of Ca^2+^-CaM/GluN1-C0 complex. Residues involved in the interactions are highlighted and labeled.

NMR spectral assignments for Ca^2+^-CaM bound to GluN1-C0 peptide were previously deposited to Biological Magnetic Resonance Bank (accession number 51715) (31). Using these previous NMR assignments, we identified the residue-specific interactions between CaM and GluN1-C0 peptide and obtained intermolecular distance restraints from nuclear overhauser effect spectroscopy (NOESY) data (Fig. 3*A*). The aromatic ring protons assigned to W858 and F852 of the GluN1-C0 peptide showed strong intermolecular NOESY crosspeaks with side chain methyl resonances assigned to CaM N-lobe (A16, I28, L33, M37, M52, I53, I64, and M72) and C-lobe (A89, V92, I101, L106, V109, M110, L113, and M146) residues, respectively. A total of 60 intermolecular NOESY distance restraints were assigned to Ca^2+^-CaM N-lobe and C-lobe residues. Three-dimensional structures of Ca^2+^-CaM bound to the GluN1-C0 peptide (PDB ID: 9PQH) were calculated using the observed NOESY data as restraints for molecular docking using HADDOCK (32). The 10 structures with lowest HADDOCK score (Fig. 3*B*) and structural statistics (Table 2) displayed convergence, with a backbone RMSD of 1.12 ± 0.12 Å. The quality of the structures was assessed using the MolProbity website (33), which shows that 97% of the residues occur in the allowed or favorable regions and no residue falls in the outlier region from the Ramachandran plot. The MolProbity score was 2.56. The binding interface of Ca^2+^-CaM/GluN1-C0 complex contains mostly hydrophobic residues (Fig. 3*C*). Importantly, GluN1 residues (A851, A854, V855, W858, and L862) contact CaM N-lobe residues (A16, F20, I28, L33, V36, M37, L40, M52, V56, I64, and M72) whereas GluN1 residues (M848, F852, and A853) contact CaM C-lobe residues (A89, V92, F93, I101, L106, V109, M110, L117, M125, M145, and M146).

**Table 2:** Statistics of NMR structures of Ca^2+^-CaM bound to *N*-methyl-D-aspartate receptor peptides

	Ca^2+^-CaM/GluN1-C0	Ca^2+^-CaM C-lobe/GluN2A-C0
HADDOCK score	−159.4 ± 1.7	−75.4 ± 2.8
Cluster size	200	200
RMSD from lowest-energy structure (Å)	0.8 ± 0.5	0.8 ± 0.5
van der Waals energy (kcal/mol)	−93.5 ± 3.0	−39.9 ± 3.6
Electrostatic energy (kcal/mol)	−387.5 ± 23.8	−176.6 ± 8.7
Restraints violation energy (kcal/mol)	20.0 ± 0.7	7.5 ± 0.4

### NMR-derived structure of Ca^2+^-CaM bound to GluN2A-C0 peptide

**Figure 4:**
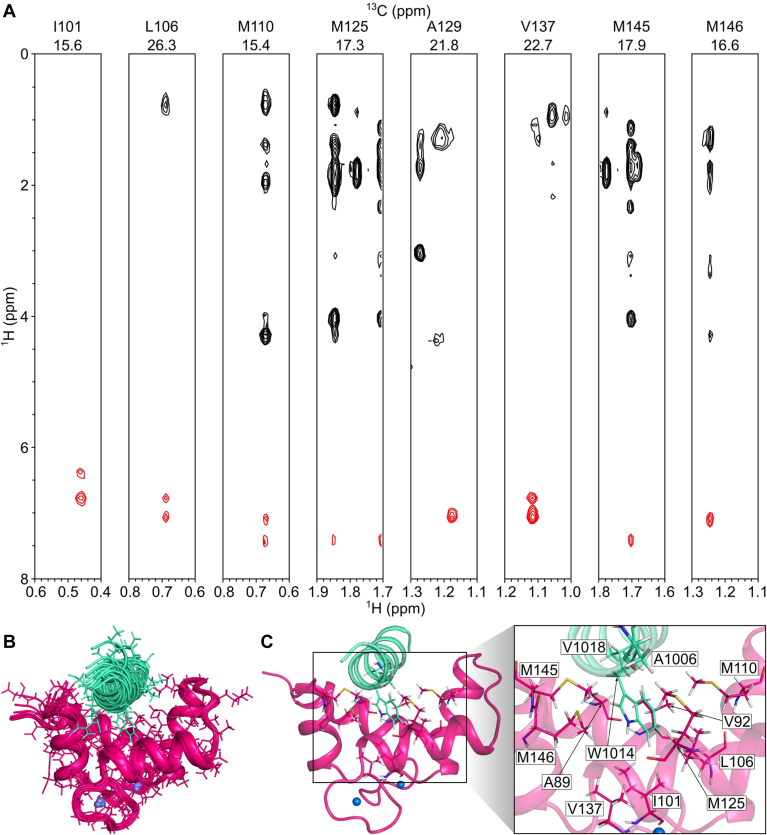
**Intermolecular interactions between Ca^2+^-CaM C-lobe and GluN2A-C0 peptide.***A*, ^13^C-edited (F2) and ^13^C-filtered (F1) nuclear overhauser effect spectroscopy strip plots of ^13^C, ^15^N-labeled Ca^2+^-CaM bound to unlabeled GluN2A-C0 peptide. Aromatic ring protons assigned to W1014 are colored *red*. *B*, overview of the 10 best structures of the Ca^2+^-CaM C-lobe (in *magenta*) bound to GluN2A-C0 (*in green*) based on HADDOCK score. Ca^2+^ ions are colored in *blue spheres*. Residues in the lowest HADDOCK score structure were highlighted in lines. *C*, *ribbon* representation of the Ca^2+^-CaM C-lobe/GluN2A-C0 complex. Residues involved in the interactions are highlighted and labeled.

NMR spectral assignments for Ca^2+^-CaM bound to the GluN2A-C0 peptide were reported previously (Biological Magnetic Resonance Bank accession number 51821) (34). These NMR assignments were used in the current study to obtain intermolecular NOESY distance restraints between Ca^2+^-CaM and the GluN2A-C0 peptide (Fig. 4*A*). The aromatic ring protons assigned to W1014 of the GluN2A-C0 peptide exhibited strong intermolecular NOESY crosspeaks with side chain methyl resonances assigned to CaM residues I101, L106, M110, M125, A129, V137, M145, and M146, indicating these atoms are less than 5 Å away from the aromatic ring of W1014. A total of 36 intermolecular NOESY crosspeaks were assigned to residues from the CaM C-lobe while no detectable intermolecular NOESY crosspeaks were identified between CaM N-lobe and GluN2A-C0. As a result, the NMR data defined the structure of the Ca^2+^-CaM C-lobe bound to GluN2A-C0. Three-dimensional structures of the Ca^2+^-CaM C-lobe bound to the GluN2A-C0 peptide (PDB ID: 9PQI) were calculated on the basis of the observed NOESY data (Fig. 4*A*) that served as restraints for molecular docking using HADDOCK (32). The 10 structures with lowest HADDOCK score (Fig. 4*B*) and structural statistics (Table 2) reveal an RMSD of 1.01 ± 0.07 Å. The quality of the structures was assessed using MolProbity (33), which shows that more than 98% of the residues occur in the allowed or favorable regions and no residue falls in the outlier region from the Ramachandran plot. The MolProbity score was 2.21. The binding interface of the Ca^2+^-CaM C-lobe/GluN2A-C0 complex contains hydrophobic GluN2A residues (A1006, W1014, and V1018) that contact CaM C-lobe residues (A89, V92, F93, I101, L106, M110, M125, V137, M145, and M146; see Fig. 4*C*).

### Mutagenesis of GluN1 residues (F852 and W858) that interact with CaM

The NMR structures of Ca^2+^-CaM bound to GluN1-C0 (Fig. 3*C*) reveal that GluN1 residues F852 and W858 point toward the CaM C-lobe and N-lobe, respectively. Exposed hydrophobic residues in the Ca^2+^-CaM N-lobe contact the aromatic ring of W858, while the Ca^2+^-CaM C-lobe contacts the aromatic ring of F852. We next determined if the GluN1 mutations F852E and W858E would weaken binding to the Ca^2+^-CaM C-lobe and N-lobe, respectively. We predicted that substitution of negatively charged glutamic acid for the bulkier, nonpolar phenylalanine and tryptophan residues would weaken binding *via* both electrostatic repulsion and steric disruption. Indeed, the ITC isotherm for GluN1-C0^W858E^ binding to full-length Ca^2+^-CaM revealed weakened binding (K_D_ = 5.5 ± 1 μM, Fig. S1*D* and Table 1) compared to that of WT GluN1-C0. The ITC isotherm for Ca^2+^-CaM N-lobe binding to GluN1-C0^W858E^ (Fig. S1*E*) failed to detect a saturating heat signal, consistent with a lack of binding. The ITC isotherm for Ca^2+^-CaM C-lobe binding to GluN1-C0^W858E^ revealed a K_D_ = 9.2 ± 2 μM (Table 1 and Fig. S1*F*), suggesting that the isolated CaM C-lobe binds with relatively low affinity to the F852 side of GluN1-C0. However, binding of intact CaM may involve rapid and ordered binding of the N-lobe to the W858 side of WT GluN1-C0, which could possibly facilitate the subsequent binding of the CaM C-lobe to the F852 side of GluN1-C0 (see SI Discussion). These findings indicate that W858E dramatically weakens GluN1-C0 binding to both full-length CaM and the N-lobe as expected from the NMR structure (Fig. 3*C*).

The ITC titrations of GluN1-C0^F852E^ peptide binding to separate N-lobe and C-lobe constructs (Table 1 and Fig. S1, *B* and *C*) exhibited *K*_D_ values of 0.40 ± 0.05 μM and 0.08 ± 0.01 μM (Table 1), respectively, indicating that GluN1-C0^F852E^ retains high affinity binding to the C-lobe. This suggests the C-lobe alone (in the absence of N-lobe) might artificially bind to the W858 side of the GluN1-C0 helix (see SI Discussion). If so, then GluN1-C0^F852E^ binding to full-length CaM is expected to form a 2:1 complex in which the N-lobe and C-lobe from a single CaM are bound to two separate peptides. Consistent with this prediction, the ITC isotherm of GluN1-C0^F852E^ binding to full-length CaM does indeed show formation of a 2:1 complex with a K_D_ = 0.28 ± 0.1 μM and N = 1.8 ± 0.3 (Table 1 and Fig. S1*A*). The 2:1 stoichiometry is also consistent with the ΔH value measured for CaM binding to the GluN1-C0^F852E^ peptide (ΔH = −16.9 kcal/mol), which is nearly twice that of CaM binding to WT GluN1-C0 (ΔH = −10.8 kcal/mol). In summary, the F852E mutation does not affect N-lobe binding, as expected from the NMR structure. However, the F852E mutation causes the C-lobe (in the absence of N-lobe) to bind artificially to the W858 side of the GluN1-C0 helix, which could explain why full-length Ca^2+^-CaM binds to two GluN1-C0^F852E^ peptides (see SI Discussion).

### GluN2A residue W1014 is essential for CaM C-lobe binding

The NMR structure of Ca^2+^-CaM bound to GluN2A-C0 revealed GluN2A residue W1014 points toward the CaM C-lobe (Fig. 4*C*), facilitating CaM C-lobe interactions with the aromatic ring of W1014. Therefore, the W1014E mutation in GluN2A is predicted to weaken GluN2A-C0 binding to the CaM C-lobe. ITC isotherms of GluN2A-C0^W1014E^ binding to the full-length Ca^2+^-CaM and Ca^2+^-CaM C-lobe exhibited exothermic heat signals that did not saturate (Fig. S1, *G* and *H*), consistent with a *K*_D_ that may be greater than the GluN2A-C0 concentration in the ITC sample cell (*K*_D_ > 30 μM). These observations suggest that GluN2A-C0 binds to the Ca^2+^-CaM C-lobe, with GluN2A residue W1014 serving as a linchpin.

### Electrophysiological studies reveal GluN1 and GluN2A mutants each disrupt CDD

**Figure 5:**
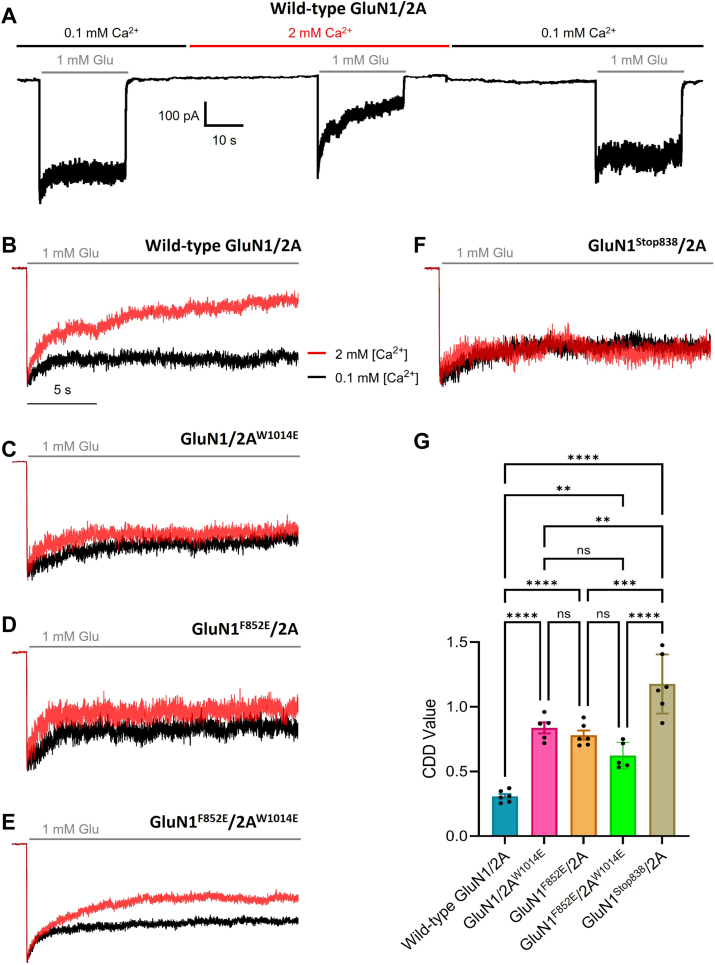
**GluN2A^W1014E^, GluN1^F852E^, and GluN1^Stop838^ mutations disrupt NMDAR CDD.***A*, example of the protocol used to measure CDD. Recording from a tsA201 cell expressing WT GluN1/2A receptors. Times of application of 1 mM Glu (*gray bars*) and of indicated external [Ca^2+^] (*black and red bars*) are shown. *B*–*F*, example traces showing overlay of the average of the initial and final responses to Glu application in 0.1 mM Ca^2+^ (*black*) and the response to Glu application in 2 mM Ca^2+^ (*red*), normalized to peak current. Because currents are normalized, current scale bars are not shown. Recordings are from cells expressing (*B*) WT GluN1/2A, (*C*) GluN1/2A^W1014E^, (*D*) GluN1^F852E^/2A, (*E*) GluN1^F852E^/2A^W1014E^, and (*F*) GluN1^Stop838^/2A receptors. Recordings shown in (*A*) and (*B*) are from the same Glu applications to the same cell. *G*, summary of CDD Values. Lower CDD Values represent greater CDD (see Experimental procedures). Data are plotted as mean ± SEM (WT GluN1/2A, 0.31 ± 0.02, n = 6; GluN1/2A^W1014E^, 0.84 ± 0.04, n = 5; GluN1^F852E^/2A, 0.78 ± 0.03, n = 6; GluN1^F852E^/2A^W1014E^, 0.62 ± 0.04, n = 5; GluN1^Stop838^/2A, 1.18 ± 0.09, n = 6). CDD Values compared with one-way ANOVA (F (4, 23) = 35.53, *p* < 0.0001) and Tukey *post hoc* test (∗∗, *p* ≤ 0.01; ∗∗∗, *p* ≤ 0.001; ∗∗∗∗, *p* ≤ 0.0001). The 5-s time scale bar shown in (*B*) also applies to (*C*–*F*). CDD, Ca^2+^-dependent channel desensitization.

To assess the functional importance of the GluN1-C0 and GluN2A-C0 regions to NMDAR CDD, GluN1^F852E^ and GluN2A^W1014E^ mutants were generated and the effects of those mutations examined electrophysiologically. We measured CDD of WT, GluN1^F852E^/2A, GluN1/2A^W1014E^, and GluN1^F852E^/2A^W1014E^ receptors to evaluate the effects of the mutations (Fig. 5, *C*–*E*).

CDD was quantified using whole-cell patch-clamp recordings from tsA201 cells transfected to express WT or mutant NMDARs. We measured the steady-state to peak ratios of Glu-activated currents in 0.1 mM external Ca^2+^ (a concentration too low to induce CDD (10, 35); and in 2 mM external Ca^2+^ to calculate the CDD Value (see Experimental procedures). Lower CDD Values reflect greater CDD. To quantify maximal CDD we measured the CDD Value of GluN1/2A (WT) receptors (Fig. 5*B*). To quantify minimal CDD we measured the CDD Value of GluN1^Stop838^/2A receptors (Fig. 5*F*); the GluN1^Stop838^ mutant, in which the GluN1 CTD was truncated at residue 838, eliminates CDD (15, 36).

In agreement with NMR and ITC data, GluN1^F852E^/2A, GluN1/2A^W1014E^, and GluN1^F852E^/2A^W1014E^ receptors all exhibited significantly reduced CDD compared to GluN1/2A. There was no significant difference between the CDD Values of GluN1^F852E^/2A, GluN1/2A^W1014E^, or of double mutant GluN1^F852E^/2A^W1014E^ receptors. The observation that there was not an additive effect of coexpressing both mutant subunits suggests that each single mutation reduces CDD *via* the same mechanism. However, the observation that the effects of the two mutations was not additive cannot be explained by a ceiling effect (it cannot be the case that either single mutation fully eliminated CDD): the CDD Values of GluN1^F852E^/2A, GluN1/2A^W1014E^, and GluN1^F852E^/2A^W1014E^ receptors were all significantly lower (CDD significantly greater) than the CDD Value of GluN1^Stop838^/2A receptors, in which GluN1 CTD truncation fully eliminates CDD. Possible explanations for why CDD of GluN1^F852E^/2A, GluN1/2A^W1014E^, and GluN1^F852E/2AW1014E^ receptors was not fully eliminated include: (a) the single residue mutations did not fully abolish Ca^2+^-CaM binding and (b) CDD may be mediated by multiple mechanisms, some of which may not depend on CaM (10, 35, 37).

The mean current amplitudes recorded from the five NMDAR constructs tested (Fig. 5*G*) were not identical. We were concerned that some of the NMDAR mutants may have exhibited reduced CDD as a result of reduced NMDAR-mediated Ca^2+^ influx rather than a direct effect on CDD. To address this possibility, we performed Pearson correlation analyses to determine whether CDD Values were correlated with peak current amplitudes in 2 mM Ca^2+^ (Fig. S2). None of the five data sets in Figure 5*G* exhibited a significant dependence of CDD Value on current amplitude, supporting the conclusion that the mutations acted through a direct effect on CDD.

## Discussion

### Structural basis of CaM binding to GluN1-C0

We present ITC (Figs. 2 and S1) and NMR structural analysis of Ca^2+^-CaM binding to GluN1-C0 (Fig. 3). The NMR structure reveals the Ca^2+^-CaM C-lobe and N-lobe bind to opposite sides of the helical GluN1-C0. The N-lobe is anchored to W858 as confirmed by the mutation W858E that weakens GluN1-C0 binding to the N-lobe (Fig. S1 and Table 1). The C-lobe is anchored to F852 in the NMR structure, but the GluN1-C0^F852E^ mutant retains high affinity binding to the isolated Ca^2+^-CaM C-lobe (in the absence of the N-lobe). This likely represents artificial binding of the C-lobe to W858 in GluN1-C0^F852E^ peptide, and therefore may explain why full-length CaM binds to two molecules of GluN1-C0^F858E^ (Fig. S1*A* and Table 1). The binding of two GluN1-C0^F852E^ to Ca^2+^-CaM (N-lobe and C-lobe each bound to a separate GluN1-C0^F852E^) is in stark contrast to the 1:1 stoichiometry observed for Ca^2+^-CaM binding to WT GluN1-C0 peptide (Fig. 2*B*). The 1:1 stoichiometry for CaM binding to WT GluN1-C0 suggests that the N-lobe may bind first to W858 (to block C-lobe binding), which could facilitate subsequent binding of the C-lobe with F852 as seen in the NMR structure (see SI Discussion). Our electrophysiological studies reveal that the F852E mutation in full-length GluN1 causes a greater than 2-fold increase in the CDD Value (representing a decrease in CDD; Fig. 5), demonstrating that Ca^2+^-CaM interaction with F852 is important for CDD. A previous study showed that mutation of GluN1^W858A^ decreases CDD (15), suggesting that Ca^2+^-CaM interaction with W858 is also important for CDD. The NMR structure of the Ca^2+^-CaM/GluN1-C0 complex (Fig. 3*C*) is similar to the X-ray structure of Ca^2+^-CaM bound to the IQ motif of the L-type calcium channel, Ca_V_1.2 (Fig. S3*A*) (38) and the NMR structure of Ca^2+^-CaM bound to the olfactory cyclic-nucleotide gated channel, CNGA2 (Fig. S3*C*) (39). Conserved hydrophobic residues in Ca_V_1.2 (F1666 and F1670), CNGA2 (F68, V72, and V75), and GluN1 (A851, A854, and W858) each contact the same residues in the CaM N-lobe (Fig. S3, *B* and *D*). Conserved hydrophobic residues in CaV1.2 (I1672, F1676, and F1769), CNGA2 (L74, V77, and W81), and GluN1 (M848, F852, and V855) contact the same residues in the CaM C-lobe.

### Structural basis of CaM binding to GluN2A-C0

Our NMR structure of Ca^2+^-CaM bound to the GluN2A-C0 peptide (Fig. 4) reveals the Ca^2+^-CaM C-lobe is anchored to W1014 in GluN2A. This contact was confirmed by the W1014E mutation, which significantly weakens GluN2A-C0 binding to both full-length Ca^2+^-CaM (Fig. S1*G*) and the Ca^2+^-CaM C-lobe (Fig. S1*H*). GluN2A-C0 has hydrophobic residues on one side of its helix (W1014 and V1018) that contact exposed hydrophobic residues in the CaM C-lobe. However, the opposite side of the GluN2A-C0 helix has hydrophilic residues (K1016, D1019, S1020), which oppose binding to exposed hydrophobic sites in CaM. Similar amphipathic CaM binding motifs from retinal cyclic-nucleotide gated channel (CNGB1) (40), creatine kinase (CK) (41), and estrogen receptors (42) each have amino acid sequences similar to that of GluN2A-C0 in which hydrophobic residues on one side of the helix are opposed by hydrophilic residues on the opposite side (Fig. 1*D*). Superimposing the NMR structure of Ca^2+^-CaM C-lobe bound to the GluN2A-C0 peptide onto the structures of the Ca^2+^-CaM C-lobe bound to the retinal CNGB1 CaM2 peptide (Fig. S4*A*) (40), and Ca^2+^-CaM C-lobe bound to the CK peptide (Fig. S4*C*) (41), shows significant conformational similarity: RMSD = 1.1 Å for Ca^2+^-CaM/CNGB1 vs 1.5 Å for Ca^2+^-CaM/CK. The conserved residues L1129 in the CNGB1 CaM2 peptide, F308 in the CK peptide, and W1014 in the GluN2A-C0 peptide each contact the same residues (A89, V92, V109, M125, and M145) in the CaM C-lobe (Fig. S4, *B* and *D*).

### A structural mechanism for Ca^2+^-dependent channel desensitization

**Figure 6:**
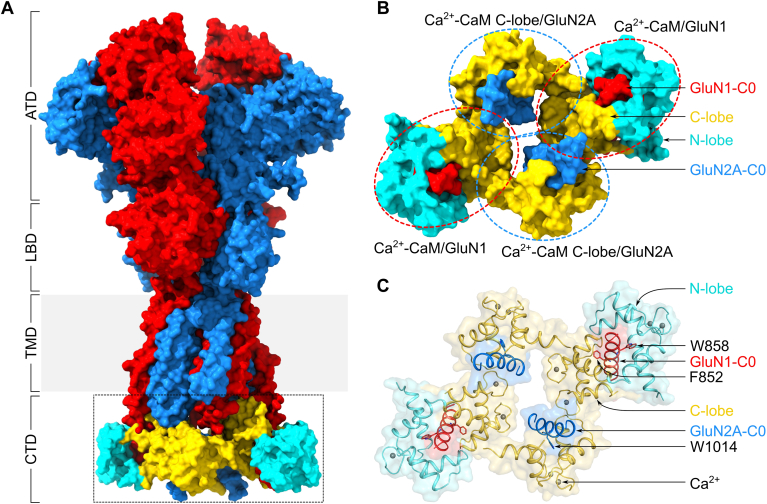
**Model of NMDAR desensitization by Ca^2+^-CaM.***A*, a surface representation of NMDAR tetramer (GluN1 in *red* and GluN2A in *blue*) bound to four Ca^2+^-CaM (N-lobe in *cyan* and C-lobe in *yellow*). The C0 regions of GluN1 and GluN2A with the four Ca^2+^-CaMs bound are highlighted in a *black* box. The structural model of the NMDAR tetramer with intact C0 sites was generated using AlphaFold3 (43). NMR structures (Figs. 3*C* and 4*C*) were superimposed on the C0 sites in the NMDAR tetramer. Finally, the structure of NMDAR bound to CaM was energy minimized using MD simulations (see SI Method and Fig. S5). *B*, a zoomed in view of the C0 regions bound to CaM. Each subunit bound to CaM is highlighted with a colored *circle*. *C*, cartoon representation showing the interactions between the GluN1/2A C0 region and CaM. GluN1-C0 residue F852 is anchored to Ca^2+^-CaM C-lobe (*yellow*) and residue W858 is anchored to Ca^2+^-CaM N-lobe (*cyan*), while GluN2A-C0 residue W1014 is anchored to Ca^2+^-CaM C-lobe (*yellow*). Ca^2+^ ions are represented as *gray spheres*.

A structural model for CDD is proposed in which the ligand-bound channel, with maximal open probability at low intracellular Ca^2+^ levels, can access a desensitized channel state at high Ca^2+^ levels due to the binding of Ca^2+^-CaM (Fig. 6). On the basis of our NMR structures (Figs. 3 and 4), recent cryo-EM structures (20, 21, 22, 23, 24, 25), and AlphaFold3 (43) we constructed a structural model of the NMDAR channel tetramer in the desensitized channel state bound to Ca^2+^-CaM (Fig. 6). A total of four CaM molecules are bound per channel tetramer with each GluN1 CaM binding site (red helix in Fig. 6, *B* and *C*) bound to Ca^2+^-CaM (Fig. 3) and each GluN2A site (blue helix in Fig. 6, *B* and *C*) bound to a separate Ca^2+^-CaM C-lobe (Fig. 4). The binding of Ca^2+^-CaM to each GluN1 in the NMDAR tetramer causes an elongation of the M4 helix (GluN1 residues 810–847) to merge with the C0 helix (GluN1 residues 847–864). The four cytosolic CaM binding sites (red and blue helices in Fig. 6, *B* and *C*) are arranged in a concentric fashion close to the channel pore. These CaM-induced conformational changes are proposed to facilitate desensitization. The NMR structure of Ca^2+^-CaM bound to GluN1-C0 (Fig. 3) reveals the CaM C-lobe is anchored to GluN1 residues (M848 and F852), whereas the CaM N-lobe is anchored to W858. Previous studies suggest the GluN1 mutations, M848E and W858A (located in the CaM binding site) each perturbed CDD (14, 15), consistent with the model in Figure 6. An important prediction of our model (Fig. 6) is that CaM C-lobe anchoring to F852 in GluN1 plays a key role in CDD. Our electrophysiological studies reveal that GluN1 mutations (F852E and Stop838) and GluN2A mutation (W1014E) each significantly disrupt CDD (Fig. 5). These results demonstrate that Ca^2+^-CaM binding to GluN1 and GluN2A as depicted in our model (Fig. 6) are important for CDD. There could be additional structural features of Ca^2+^-CaM bound to the C0 sites in the intact channel tetramer that might not be seen in our structures with the C0 peptides. Future cryo-EM studies are needed to determine the structure of the NMDAR channel tetramer bound to CaM to further test the predictions of our model (Fig. 6).

## Experimental procedures

### Preparation of protein and peptide samples

The expression and purification of CaM proteins (CaM, CaM N-lobe, and CaM C-lobe) followed the method described previously (44). To express uniformly ^13^C, ^15^N-labeled CaM samples, cells were grown in M9 minimal medium supplemented with 1 g/L ^15^NH_4_Cl, and 3 g/L ^13^C-labled glucose (Cambridge Isotopes Laboratories) as the sole nitrogen and carbon sources, respectively. The WT and mutant (GluN1-C0^F852E^, GluN1-C0^W858E^, and GluN2A-C0^W1014E^) peptides of GluN1-C0 (residues 841–865) and GluN2A-C0 (residues 1004–1023) were purchased from GenScript, dissolved in DMSO-d_6_, and quantified using UV–Vis absorption spectroscopy.

### Isothermal titration calorimetry (ITC)

ITC experiments were performed using a VP-ITC calorimeter (Micro-Cal) at 27 °C. The data were acquired and processed with MicroCal software (https://www.originlab.com) as described previously (40). Samples of CaM and GluN1-C0 (or GluN2A-C0) peptide were prepared by exchanging each with buffer containing either 20 mM Tris (pH 8.0), 100 mM KCl, and 2 mM EGTA (apo-CaM) or 20 mM Tris (pH 7.0), 100 mM KCl, and 1 mM CaCl_2_ (Ca^2+^-CaM). For ITC experiments in which full-length CaM was used, the GluN1-C0 (or GluN2A-C0) peptide was the injectant (forward titration); for ITC experiments in which the isolated CaM N-lobe or C-lobe was used, the CaM lobe was the injectant (reverse titration). In the forward titration, the injectant consisted of 35 injections (7 μl each) of 0.3 mM GluN1-C0 (or GluN2A-C0). The concentration of CaM was 30 μM in 1.5 ml (sample cell). Peptide titrations with CaM N-lobe (or C-lobe) were done in reverse order with CaM lobe (injectant) and peptide (titrand).

### NMR spectroscopy

All NMR samples were prepared in 20 mM Tris-d_11_ (pH 7.0) and 1 mM CaCl_2_ containing either 8% or 100% (v/v) D_2_O. A 2.5-fold excess unlabeled peptide (GluN1-C0 or GluN2A-C0) was added to ^13^C, ^15^N-labeled Ca^2+^-CaM, incubated at room temperature for 30 min, concentrated to 0.4 mM in a final volume of 0.3 ml, and packed into Shigemi NMR tubes (Shigemi Inc). All NMR experiments were performed at 308 K on a Bruker Avance III 800 MHz spectrometer equipped with TCI cryogenic probe. 2D ^15^N-^1^H and ^13^C-^1^H HSQC spectra were recorded and assigned as described previously (31, 34). 3D and 2D ^13^C-edited and filtered NOESY experiments were performed with a mixing time of 120 ms. All NMR spectra were processed using NMRPipe (45) and SPARKY (46) was used for spectral assignment and visualization.

### Molecular docking calculations

The complex structures of Ca^2+^-CaM bound to GluN1-C0 and of Ca^2+^-CaM C-lobe bound to GluN2A-C0 were calculated by the web-based docking program HADDOCK 2.4 (32) following a previous protocol (40). The docking calculation used the crystal structures of Ca^2+^-CaM and Ca^2+^-CaM C-lobe (from PDB ID: 2F3Y (38) which were docked with the helical structure of the GluN1-C0 and GluN2A-C0 peptides, respectively. The helical structures of GluN1-C0 and GluN2A-C0 peptides were generated using Modeller 9.25 (47). 60 and 36 intermolecular distance restraints between the protein and peptide derived from the NOESY data were introduced as unambiguous restraints for the docking calculation of Ca^2+^-CaM/GluN1-C0 and Ca^2+^-CaM C-lobe/GluN2A-C0, respectively. Ca^2+^ ions were docked using restraints derived from the crystal structure of Ca^2+^-CaM bound to Ca_V_1.2 IQ domain (PDB ID: 2F3Y (38)). The docking calculation was initiated by generating 1000 structures using rigid body docking. The best 200 structures from the previous step were proceeded to a semi-flexible simulated annealing step followed by a water refinement step. The 10 structures with lowest HADDOCK score, RMSD, energy values, and Z-score were deposited to RCSB PDB (PDB ID: 9PQH for Ca^2+^-CaM/GluN1-C0 and PDB ID: 9PQI for Ca^2+^-CaM C-lobe/GluN2A-C0). The structure quality was assessed by PROCHECK-NMR (48) and MolProbity (33). The coordinate file with the lowest HADDOCK score was chosen for the final structural model displayed in this study.

### Cell culture, receptor constructs, mutagenesis, and transfection

Electrophysiological experiments were conducted on tsA201 cells (Millipore Sigma Cat # 96121229-1VL). Cells were maintained in Dulbecco’s Modified Eagle Medium (DMEM) enriched with 10% fetal bovine serum and 1% GlutaMAX (Thermo Fisher Scientific) as previously described (49). Cells were transfected with complementary DNAs (cDNAs) encoding WT or mutant rat GluN1-1a (GluN1; GenBank accession number X63255, in pcDNA3.1) and GluN2A (accession number M91561, in pcDNA1) NMDAR subunits. Site-directed mutagenesis was performed using the QuikChange XL Site-Directed Mutagenesis Kit (Stratagene) to generate the GluN1^F852E^ and GluN2A^W1014E^ mutant subunits. Mutagenized NMDAR subunit cDNAs from isolated colonies were sequenced from 100 to 200 bases upstream to 100 to 200 bases downstream of each mutation (Genewiz). cDNA encoding the GluN1 subunit with a stop codon at residue 838, the beginning of the C-terminal domain (GluN1^Stop838^) was a kind gift from Dr Gary Westbrook (37). The constructs pCI-neo: enhanced green fluorescent protein (eGFP):GluN1 (50) and eGFP:pIRES:GluN2A, both of which allowed expression of an NMDAR subunit and eGFP as separate proteins, were kind gifts from Dr Kasper Hansen.

Transient transfections were carried out using FuGENE 6 (Promega). Cells were transfected to express WT or mutant GluN1 and GluN2A subunits, WT calmodulin (CaM, in pcDNA3), and eGFP (by transfection with pCI-neo:eGFP:GluN1, eGFP:pIRES:GluN2A, or eGFP (Genbank ACS32473) in pRK7) to identify transfected cells. Plasmids encoding the following cDNA combinations were transfected at the indicated ratios: (a) (GluN1 or GluN1^F852E^ or GluN1^Stop838^) and (GluN2A or GluN2A^W1014E^) and (CaM) and (eGFP) at a 1:2:3:1 ratio; (b) (pCI-neo:eGFP:GluN1) and (GluN2A or GluN2A^W1014E^) and (CaM) at a 1:2:3 ratio; (c) (GluN1 or GluN1^F852E^ or GluN1^Stop838^) and (eGFP:pIRES:GluN2A) and (CaM) at a 1:2:3 ratio. To prevent NMDAR-mediated excitotoxicity following transfection, the competitive antagonist D, L-2-amino-5-phosphonopentanoate (200 μM) was added to the culture medium.

### Patch-clamp electrophysiology and data analysis

24 to 48 h after transfection, patch-clamp recordings were conducted in the whole-cell voltage-clamp configuration. Patch pipettes were fabricated from borosilicate capillary tubing (Sutter Instruments, OD = 1.5 mm, ID = 0.86 mm) using a Flaming Brown P-97 electrode puller (Sutter instruments), and fire-polished to an open-tip resistance of 2 to 8 MΩ. Intracellular (pipette) solution contained (in mM): 128 Cs-Gluconate, 12 CsCl, 5 EGTA, 10 HEPES, and 4 MgATP, adjusted to pH 7.2 ± 0.05 with CsOH. Osmolality was 277 mOsm. eGFP-positive cells were identified *via* epifluorescence on an inverted Zeiss microscope. Cells were voltage-clamped at −72 mV after correction for a liquid junction potential of −13 mV. The external recording solution contained (in mM): 140 NaCl, 2.8 KCl, either 0.1 or 2 CaCl_2_, 10 HEPES, and 0.01 EDTA; pH was adjusted to 7.2 ± 0.05 with NaOH and osmolality was adjusted to 290 ± 10 mOsm using sucrose. 100 μM glycine was included in all external solutions. Whole-cell currents were evoked by rapid application of 1 mM Glu using an in-house fast perfusion system (51). Recordings were performed using an Axopatch 200B amplifier (Molecular Devices), low-pass filtered at 5 kHz and sampled at 20 kHz using a Digidata 1440A digitizer (Molecular Devices), and Clampex 10.2 software (Molecular Devices). Cells were excluded from analysis if any of the following criteria were met: series resistance >20 MΩ; peak glutamate-evoked current >2.5 nA in 2 mM Ca^2+^; a >20% difference in steady-state to peak current ratio between measurements during the first and last Glu application in 0.1 mM Ca^2+^; holding current more negative than −200 pA; baseline drift >100 pA; membrane potential drift >2 mV. Series resistance was compensated ≥80% using the amplifier’s prediction and correction controls. Data were analyzed using Clampfit versions 10.3 or 10.7 (Molecular Devices; https://www.moleculardevices.com/products/axon-patch-clamp-system/acquisition-and-analysis-software/pclamp-software-suite), GraphPad Prism (versions 7–9; https://www.graphpad.com/), or Origin 9.3 (OriginLab). To measure CDD, the following application protocol was used (Fig. 5*A*): Glu was applied for 20 s in the 0.1 mM Ca^2+^ external solution; 45 s later Glu was applied for 20 s in the 2 mM Ca^2+^ external solution; 45 s later Glu again was applied for 20 s in the 0.1 mM Ca^2+^ solution. Steady-state to peak current ratios (I_ss_/I_peak_) were calculated by dividing the steady-state current (averaged over a 1 s window at the end of a Glu application) by the peak current (average current during a 30 ms window centered on the peak response to Glu application). The I_ss_/I_peak_ value from the first and the last Glu applications in the 0.1 mM Ca^2+^ external solution were averaged. All current values were baseline-subtracted prior to analysis. The CDD Value was calculated as:

$$\text{CDD}\;\text{Value}=\left(I_{\text{ss}}/I_{\text{peak}}\;\text{in}\;2\;\text{mM}\;{\text{Ca}}^{2+}\right)\;/\;\left(\text{average}\;I_{\text{ss}}/I_{\text{peak}}\;\text{in}\;0.1\;\text{mM}\;{\text{Ca}}^{2+}\right)$$

Statistical analyses were conducted using GraphPad Prism (versions 7–9). One-way ANOVAs followed by Tukey’s *post hoc* tests were used as indicated. In Figure 5, *n* refers to the number of individual cells recorded. Mean CDD Values represent the average across all cells, and all error bars indicate ± standard error of the mean (SEM).

## Data availability

The data used to support the findings of this study are contained within the manuscript. The atomic coordinates were deposited in the Protein Data Bank (PDB ID: 9PQH for Ca^2+^-CaM/GluN1-C0 and PDB ID: 9PQI for Ca^2+^-CaM C-lobe/GluN2A-C0).

## Supporting information

This article contains supporting information (38, 39, 40, 41, 43, 52).

## Conflict of interest

The authors declare that they have no conflicts of interest with the contents of this article.

## References

[1] Bliss T.V.P., Collingridge G.L. (1993). A synaptic model of memory: long-term potentiation in the hippocampus. Nature.

[2] Tang Y.-P., Shimizu E., Dube G.R., Rampon C., Kerchner G.A., Zhuo M. (1999). Genetic enhancement of learning and memory in mice. Nature.

[3] Paoletti P., Bellone C., Zhou Q. (2013). NMDA receptor subunit diversity: impact on receptor properties, synaptic plasticity and disease. Nat. Rev. Neurosci..

[4] Iacobucci G.J., Popescu G.K. (2017). NMDA receptors: linking physiological output to biophysical operation. Nat. Rev. Neurosci..

[5] Benveniste M., Mayer M.L. (1991). Kinetic analysis of antagonist action at N-methyl-D-aspartic acid receptors. Two binding sites each for glutamate and glycine. Biophys. J..

[6] Monyer H., Sprengel R., Schoepfer R., Herb A., Higuchi M., Lomeli H. (1992). Heteromeric NMDA receptors: molecular and functional distinction of subtypes. Science.

[7] Karakas E., Furukawa H. (2014). Crystal structure of a heterotetrameric NMDA receptor ion channel. Science.

[8] Lee C.H., Lü W., Michel J.C., Goehring A., Du J., Song X. (2014). NMDA receptor structures reveal subunit arrangement and pore architecture. Nature.

[9] Umemiya M., Chen N., Raymond L.A., Murphy T.H. (2001). A calcium-dependent feedback mechanism participates in shaping single NMDA miniature EPSCs. J. Neurosci..

[10] Krupp J.J., Vissel B., Heinemann S.F., Westbrook G.L. (1996). Calcium-dependent inactivation of recombinant N-methyl-D-aspartate receptors is NR2 subunit specific. Mol. Pharmacol..

[11] Rosenmund C., Feltz A., Westbrook G.L. (1995). Calcium-dependent inactivation of synaptic NMDA receptors in hippocampal neurons. J. Neurophysiol..

[12] Ehlers M.D., Zhang S., Bernhardt J.P., Huganir R.L. (1996). Inactivation of NMDA receptors by direct interaction of calmodulin with the NR1 subunit. Cell.

[13] Akyol Z., Bartos J.A., Merrill M.A., Faga L.A., Jaren O.R., Shea M.A. (2004). Apo-calmodulin binds with its C-terminal domain to the N-methyl-D-aspartate receptor NR1 C0 region. J. Biol. Chem..

[14] Zhang S., Ehlers M.D., Bernhardt J.P., Su C.T., Huganir R.L. (1998). Calmodulin mediates calcium-dependent inactivation of N-methyl-D-aspartate receptors. Neuron.

[15] Iacobucci G.J., Popescu G.K. (2017). Resident calmodulin primes NMDA receptors for Ca2+-dependent inactivation. Biophys. J..

[16] Ataman Z.A., Gakhar L., Sorensen B.R., Hell J.W., Shea M.A. (2007). The NMDA receptor NR1 C1 region bound to calmodulin: structural insights into functional differences between homologous domains. Structure.

[17] Wang C., Wang H.-G., Xie H., Pitt G.S. (2008). Ca2+/CaM controls Ca2+-dependent inactivation of NMDA receptors by dimerizing the NR1 C termini. J. Neurosci..

[18] Ehlers M.D., Tingley W.G., Huganir R.L. (1995). Regulated subcellular distribution of the NR1 subunit of the NMDA receptor. Science.

[19] Bajaj G., Hau A.M., Hsu P., Gafken P.R., Schimerlik M.I., Ishmael J.E. (2014). Identification of an atypical calcium-dependent calmodulin binding site on the C-terminal domain of GluN2A. Biochem. Biophys. Res. Commun..

[20] Zhu S., Stein R.A., Yoshioka C., Lee C.-H., Goehring A., Mchaourab H.S. (2016). Mechanism of NMDA receptor inhibition and activation. Cell.

[21] Lü W., Du J., Goehring A., Gouaux E. (2017). Cryo-EM structures of the triheteromeric NMDA receptor and its allosteric modulation. Science.

[22] Zhang J.B., Chang S., Xu P., Miao M., Wu H., Zhang Y. (2018). Structural basis of the proton sensitivity of human GluN1-GluN2A NMDA receptors. Cell Rep..

[23] Zhang Y., Ye F., Zhang T., Lv S., Zhou L., Du D. (2021). Structural basis of ketamine action on human NMDA receptors. Nature.

[24] Tajima N., Simorowski N., Yovanno R.A., Regan M.C., Michalski K., Gómez R. (2022). Development and characterization of functional antibodies targeting NMDA receptors. Nat. Commun..

[25] Zhang J., Zhang M., Wang Q., Wen H., Liu Z., Wang F. (2023). Distinct structure and gating mechanism in diverse NMDA receptors with GluN2C and GluN2D subunits. Nat. Struct. Mol. Biol..

[26] Hardingham G.E., Bading H. (2010). Synaptic versus extrasynaptic NMDA receptor signalling: implications for neurodegenerative disorders. Nat. Rev. Neurosci..

[27] Soto D., Altafaj X., Sindreu C., Bayés À. (2014). Glutamate receptor mutations in psychiatric and neurodevelopmental disorders. Commun. Integr. Biol..

[28] Bourgeron T. (2015). From the genetic architecture to synaptic plasticity in autism spectrum disorder. Nat. Rev. Neurosci..

[29] Balu D.T. (2016). The NMDA receptor and schizophrenia: from pathophysiology to treatment. Neuropsychopharmacol..

[30] Duman R.S., Aghajanian G.K., Sanacora G., Krystal J.H. (2016). Synaptic plasticity and depression: new insights from stress and rapid-acting antidepressants. Nat. Med..

[31] Bej A., Ames J.B. (2023). Chemical shift assignments of calmodulin bound to the GluN1 C0 domain (residues 841–865) of the NMDA receptor. Biomol. NMR Assign..

[32] Honorato R.V., Trellet M.E., Jiménez-García B., Schaarschmidt J.J., Giulini M., Reys V. (2024). The HADDOCK2.4 web server for integrative modeling of biomolecular complexes. Nat. Protoc..

[33] Williams C.J., Headd J.J., Moriarty N.W., Prisant M.G., Videau L.L., Deis L.N. (2018). MolProbity: more and better reference data for improved all-atom structure validation. Protein Sci..

[34] Bej A., Ames J.B. (2023). Chemical shift assignments of calmodulin bound to a cytosolic domain of GluN2A (residues 1004–1024) from the NMDA receptor. Biomol. NMR Assign..

[35] Legendre P., Rosenmund C., Westbrook G.L. (1993). Inactivation of NMDA channels in cultured hippocampal neurons by intracellular calcium. J. Neurosci..

[36] Krupp J.J., Vissel B., Heinemann S.F., Westbrook G.L. (1998). N-terminal domains in the NR2 subunit control desensitization of NMDA receptors. Neuron.

[37] Krupp J.J., Vissel B., Thomas C.G., Heinemann S.F., Westbrook G.L. (1999). Interactions of calmodulin and α-actinin with the NR1 subunit modulate Ca2+-dependent inactivation of NMDA receptors. J. Neurosci..

[38] Fallon J.L., Halling D.B., Hamilton S.L., Quiocho F.A. (2005). Structure of calmodulin bound to the hydrophobic IQ domain of the cardiac Cav1.2 calcium channel. Structure.

[39] Irene D., Huang J.-W., Chung T.-Y., Li F.-Y., Tzen J.T.-C., Lin T.-H. (2013). Binding orientation and specificity of calmodulin to rat olfactory cyclic nucleotide-gated ion channel. J. Biomol. Struct. Dyn..

[40] Bej A., Ames J.B. (2022). NMR structures of calmodulin bound to two separate regulatory sites in the retinal cyclic nucleotide-gated channel. Biochemistry.

[41] Sprenger J., Trifan A., Patel N., Vanderbeck A., Bredfelt J., Tajkhorshid E. (2021). Calmodulin complexes with brain and muscle creatine kinase peptides. Curr. Res. Struct. Biol..

[42] Zhang Y., Li Z., Sacks D.B., Ames J.B. (2012). Structural basis for Ca2+-induced activation and dimerization of estrogen receptor α by calmodulin. J. Biol. Chem..

[43] Abramson J., Adler J., Dunger J., Evans R., Green T., Pritzel A. (2024). Accurate structure prediction of biomolecular interactions with AlphaFold 3. Nature.

[44] Bej A., Ames J.B. (2022). Chemical shift assignments of calmodulin under standard conditions at neutral pH. Biomol. NMR Assign..

[45] Delaglio F., Grzesiek S., Vuister G., Zhu G., Pfeifer J., Bax A. (1995). NMRPipe: a multidimensional spectral processing system based on UNIX pipes. J. Biomol. NMR.

[46] Goddard T.D., Kneller D.G. (2004).

[47] Sali A., Blundell T.L. (1993). Comparative protein modelling by satisfaction of spatial restraints. J. Mol. Biol..

[48] Laskowski R.A., Rullmann J.A.C., MacArthur M.W., Kaptein R., Thornton J.M. (1996). AQUA and PROCHECK-NMR: programs for checking the quality of protein structures solved by NMR. J. Biomol. NMR.

[49] Glasgow N.G., Johnson J.W. (2014). Whole-cell patch-clamp analysis of recombinant NMDA receptor pharmacology using brief glutamate applications. Methods Mol. Biol..

[50] Yi F., Zachariassen L.G., Dorsett K.N., Hansen K.B. (2018). Properties of triheteromeric N-methyl-d-aspartate receptors containing two distinct GluN1 isoforms. Mol. Pharmacol..

[51] Glasgow N.G., Povysheva N.V., Azofeifa A.M., Johnson J.W. (2017). Memantine and ketamine differentially alter NMDA receptor desensitization. J. Neurosci..

[52] Cudia D.L., Ahoulou E.O., Bej A., Janssen A.N., Scholten A., Koch K.-W. (2024). NMR structure of retinal guanylate cyclase activating protein 5 (GCAP5) with r22a mutation that abolishes dimerization and enhances cyclase activation. Biochemistry.

