# Supplementary material for: Structural basis and functional analysis of NMDA receptor regulation by calmodulin
Source: J Biol Chem. 2026 Jan 7;302(2):111131. doi: 10.1016/j.jbc.2026.111131 (PMC12870862; doi:10.1016/j.jbc.2026.111131)
Supplement: Supporting Information [file mmc1.docx]

**Supporting Information**

**Structural Basis and Functional Analysis of NMDA Receptor Regulation by Calmodulin**

Aritra Bej^1,2*^, M. Quincy Erickson-Oberg^3*^, Aparna Nigam^3*^, Isaac Yu^1^, Johannes W. Hell^2^, Jon W. Johnson^3†^ and James B. Ames^1†^

From the ^1^Department of Chemistry, University of California, Davis, CA, ^2^Department of Pharmacology, University of California, Davis, CA, and ^3^Department of Neuroscience and Center for Neuroscience, University of Pittsburgh, Pittsburgh, PA.

^*^AB, MQE-O, and AN contributed equally to this work.

^†^To whom correspondence should be addressed. (JWJ) Department of Neuroscience and Center for Neuroscience, University of Pittsburgh, Pittsburgh, PA, USA. Telephone: (412) 624-4295, Email: [jjohnson@pitt.edu](mailto:jjohnson@pitt.edu). (JBA) Department of Chemistry, University of California, Davis, CA 95616, Tel (530) 752-6358, FAX (530) 752-8995, Email: [jbames@ucdavis.edu](mailto:jbames@ucdavis.edu).

**SI Method**

**Computational modeling of NMDAR tetramer bound to Ca^2+^-CaM.** The structural model of the NMDAR tetramer bound to Ca^2+^-CaM (Fig. 6) was generated by superimposing the NMR structures (Figs. 3C and 4C) onto a predicted structure of the NMDAR tetramer generated by AlphaFold3 (1). An initial AlphaFold3 model was prepared that included two subunits of GluN1 (residues K25 – R865), two subunits of GluN2A (residues L34 – Q1023), and four molecules of Ca^2+^-CaM (Fig. S5). This model shows that GluN1 forms a C0 helix (residues 846-864) similar to that seen in the NMR structure (Fig. 3). The GluN1-C0 helix in the model is contiguous with an elongated M4 helix (GluN1 residues 810-847). The CaM C-lobe is bound to the W858 side of the GluN1 C0 helix in the model. The F852 side of the C0 helix in the model is not bound to CaM. Thus, the AlphaFold3 model is inconsistent with our NMR structure (Fig. 3C) in which the CaM C-lobe is bound to the F852 side of the C0 helix and CaM N-lobe is bound to W858 side. The AlphaFold3 model also predicts that the CaM N-lobe is bound to a separate cytosolic helix in GluN2A (residues 890 – 910), which has not been previously characterized and is inconsistent with our NMR structures. AlphaFold3 correctly predicts that the C-lobe from a second CaM (see CaM3 colored magenta in Fig. S5B) is bound to the C0 helix of GluN2A (residues 1010 – 1018, blue in Fig. S5B), which is consistent with our NMR structure of Ca^2+^-CaM C-lobe bound to GluN2A-C0 peptide (Fig. 4C). Lastly, the AlphaFold3 model predicts that the C-lobe from two separate CaM molecules (bound to the channel tetramer) are superimposed on top of each other (see C-lobe of CaM1 and CaM3 in Fig. S5B), which is energetically not feasible. It is surprising that AlphaFold3 would generate a structural model that has overlapping atoms from two separate CaM molecules, which calls into question the validity of the bound CaM structures in the model. In summary, AlphaFold3 predicted a structure of the NMDAR tetramer that includes the C0 helix from GluN1. However, AlphaFold3 could not reliably predict the structure of the NMDAR tetramer bound to four molecules of CaM. Instead, an alternative model of the NMDAR tetramer bound to 4 Ca^2+^-CaM was generated by superimposing the NMR structure of Ca^2+^-CaM bound to GluN1-C0 peptide (Fig. 3C) onto each GluN1 C0 helix in the NMDAR tetramer from the AlphaFold3 model in Fig. S5A. Two molecules of the GluN2A C0 helix bound to the Ca^2+^-CaM C-lobe (NMR structure in Fig. 4C) were then manually positioned to form a concentric arrangement of 4 Ca^2+^-CaM bound to the NMDAR tetramer near the channel pore (Fig. 6C). The final model was energy minimized in a vacuum condition using MD simulations as described previously (2).

**SI Discussion**

**Kinetically controlled binding of the CaM N-lobe to the W858 side of GluN1-C0.** The NMR structure of Ca^2+^-CaM bound to the GluN1-C0 peptide reveals a 1:1 complex in which the N-lobe contacts W858 and C-lobe contacts F852 (Fig. 3C). However, our ITC data indicate that wild-type GluN1-C0 peptide binds the Ca^2+^-CaM C-lobe alone (K_D_ = 0.10) with higher affinity than the N-lobe alone (K_D_ = 0.58), and GluN1-C0^F852E^ peptide also binds the C-lobe (K_D_ = 0.08) with higher affinity than the N-lobe (K_D_ = 0.40; Table 1).These binding data imply the C-lobe alone binds to the W858 side of the GluN1-C0 peptide with ~4-fold higher affinity than that of the N-lobe. Also, the full-length Ca^2+^-CaM binds to GluN1-C0^F852E^ peptide with a 2:1 stoichiometry (two peptides bound per CaM), in contrast to wild-type GluN1-C0 that binds to Ca^2+^-CaM with a 1:1 stoichiometry. How can these binding data be reconciled with the NMR structure? We propose that Ca^2+^-CaM binding to the GluN1-C0 peptide is kinetically controlled in which the N-lobe binds faster than the C-lobe to the W858 side of GluN1-C0, even though the C-lobe binds to the W858 side with higher affinity. This kinetically controlled and ordered binding could explain why the N-lobe binds first to W858, which then facilitates subsequent C-lobe binding to the F852 side of the target helix as seen in the NMR structure. We suggest the Ca^2+^-CaM C-lobe binding to the F852 side of GluN1-C0 locks CaM in a stable 1:1 complex with the bound peptide. This is in contrast to full-length Ca^2+^-CaM binding to GluN1-C0^F852E^ in which the N-lobe and C-lobe each bind to the W858 side of two separate GluN1-C0^F852E^ peptides in a stable 2:1 complex (two GluN1-C0^F852E^ peptides bound per CaM).


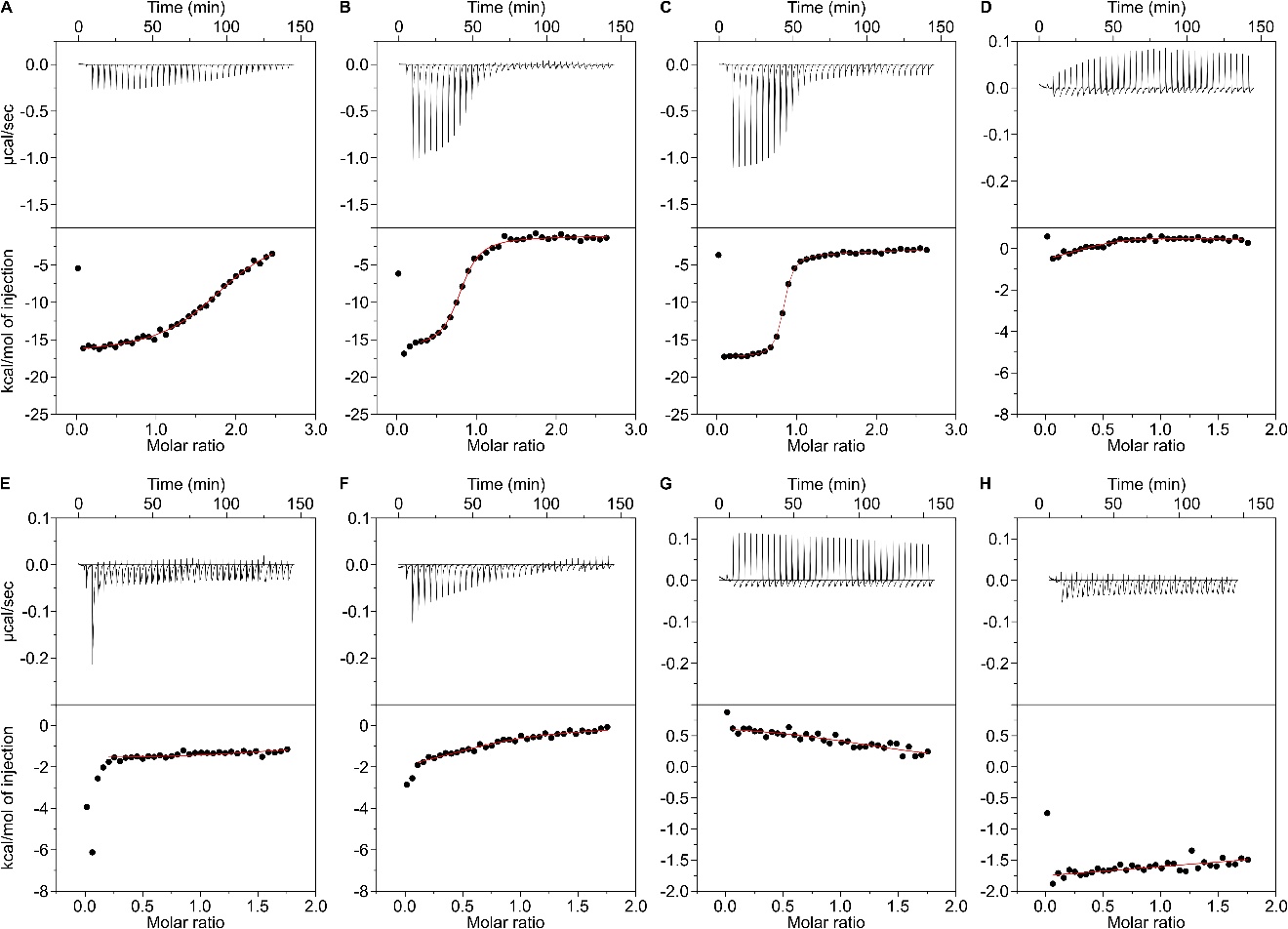


**Figure S1. Binding of CaM and CaM-lobes to NMDAR mutant peptides.** **(A) – (C)** ITC isotherms of GluN1-C0^F852E^ binding to **(A)** full-length Ca^2+^-CaM, **(B)** Ca^2+^-CaM N-lobe, and **(C)** Ca^2+^-CaM C-lobe. **(D) – (F)** GluN1-C0^W858E^ binding to **(D)** full-length Ca^2+^-CaM, **(E)** Ca^2+^-CaM N-lobe, and **(F)** Ca^2+^-CaM C-lobe. **(G) – (H)** GluN2A^W1014E^ binding to **(G)** full-length Ca^2+^-CaM and **(H)** Ca^2+^-CaM C-lobe. Each binding isotherm was fit to a one-site model and the binding parameters (Δ*H* and *K*_D_) are given in Table 1.


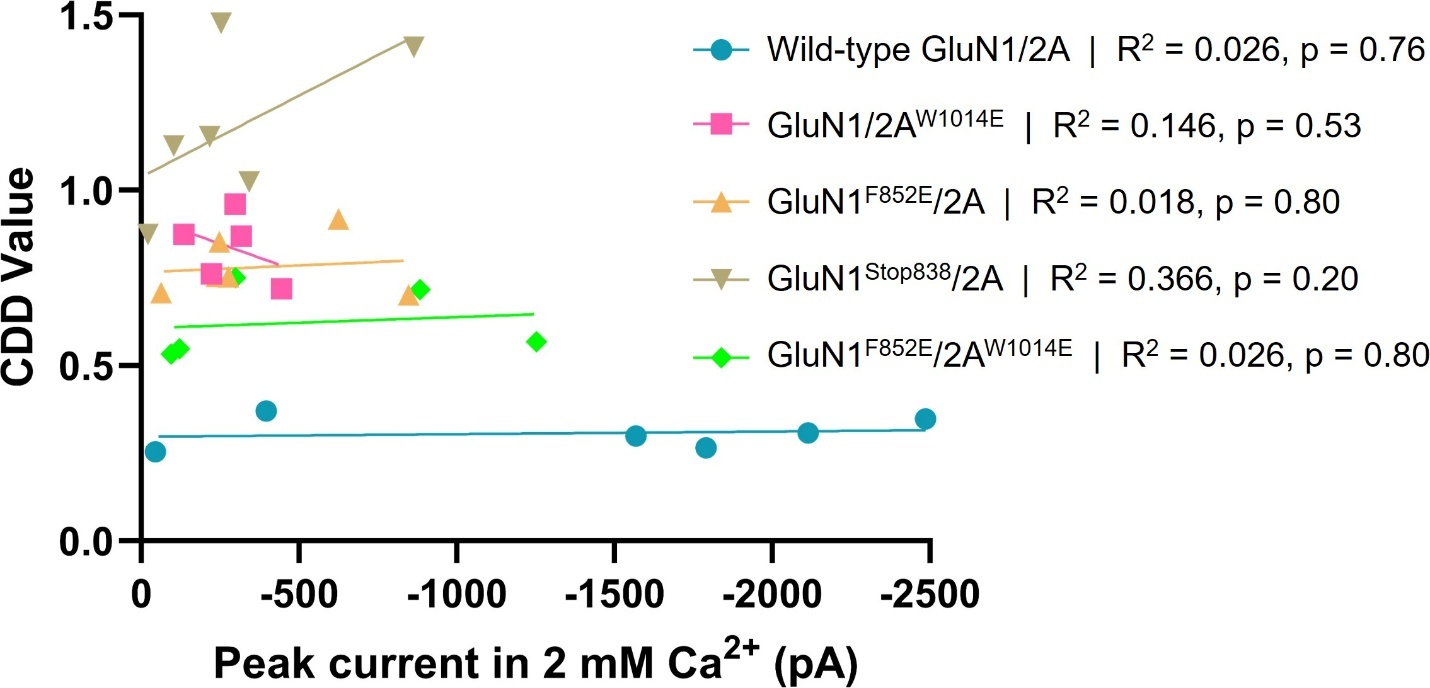


**Figure S2. Pearson correlation analysis of relation between CDD value and peak current amplitude.** Scatter plots of CDD value versus peak current amplitude (measured in 2 mM extracellular Ca^2+^) for each of the five NMDAR constructs tested (Fig. 5). Each point represents results from an independent cell recording. Linear regression fits are plotted for each construct. NMDAR constructs are identified in the figure key, which also gives corresponding R2 and p-values. None of the constructs exhibited a significant dependence of CDD on peak current amplitude, suggesting that differences in CDD among constructs are not attributable to differences in current amplitudes. Multiple linear regression analysis also indicated that peak current amplitude in 2 mM Ca2+ was not a significant predictor of CDD (p = 0.42).
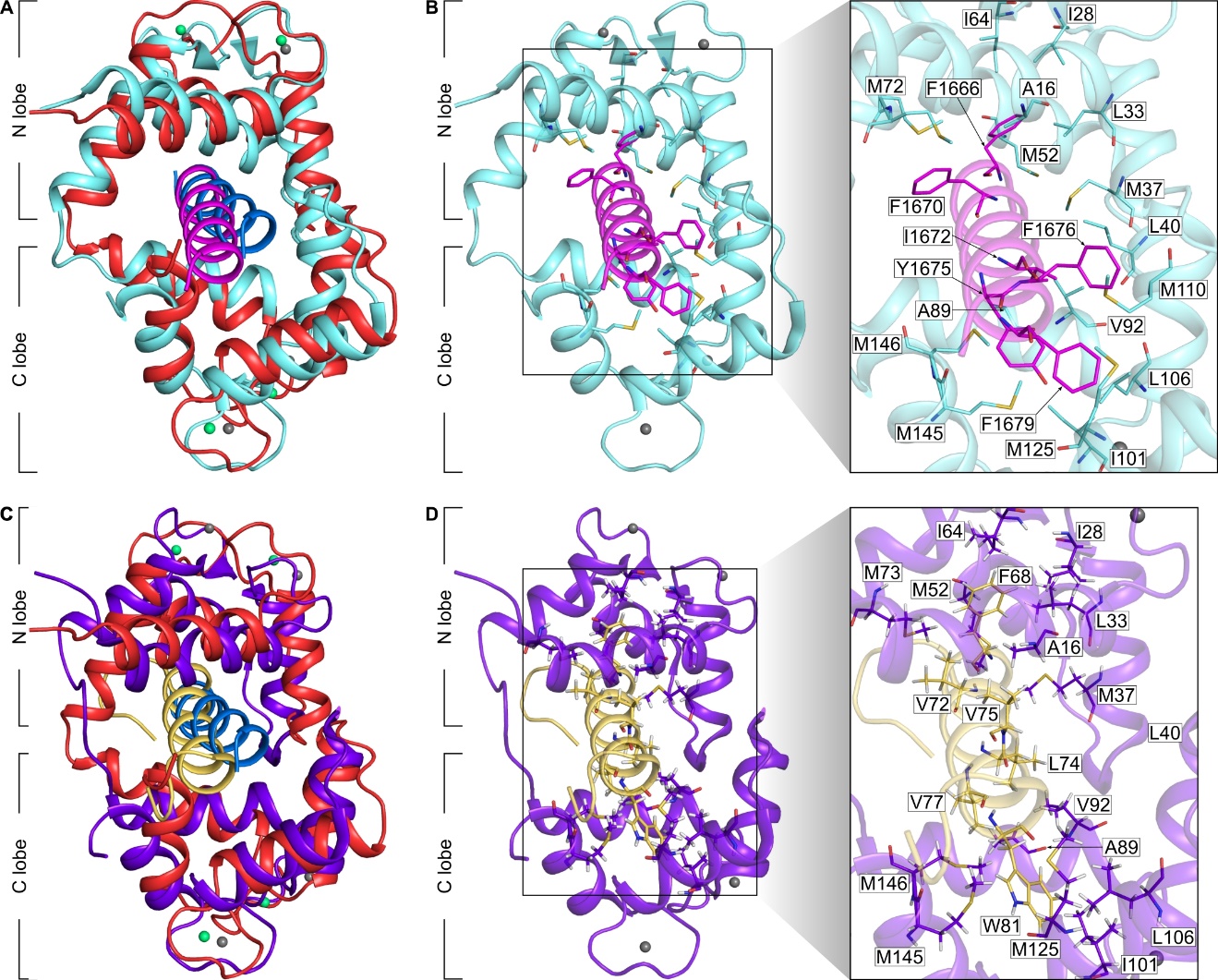


**Figure S3. Structural comparison of full-length Ca^2+^-CaM bound to various target peptides.** **(A)** NMR-derived structure of Ca^2+^-CaM (red) bound to GluN1-C0 peptide (blue) overlaid onto the X-ray structure of Ca^2+^-CaM (cyan) bound to Ca_V_1.2 IQ peptide (magenta, PDB ID: 2F3Y (3)). **(B)** Ribbon representation of Ca^2+^-CaM bound to Ca_V_1.2 IQ peptide. Expanded view of the boxed region shows residues at the binding interface highlighted as sticks and labeled. **(C)** NMR-derived structures of Ca^2+^-CaM (red) bound to GluN1-C0 peptide (blue) overlaid onto Ca^2+^-CaM (purple) bound to CNGA2 peptide (yellow, PDB ID: 2M0K (4)). **(D)** Ribbon representation of Ca^2+^-CaM bound to CNGA2 peptide. Expanded view of the boxed region shows residues at the binding interface highlighted as sticks and labeled. Ca^2+^ ions are colored green (for Ca^2+^-CaM bound to GluN1-C0 peptide) or gray (for Ca^2+^-CaM bound to CaV1.2 IQ peptide and Ca^2+^-CaM bound to CNGA2 peptide).


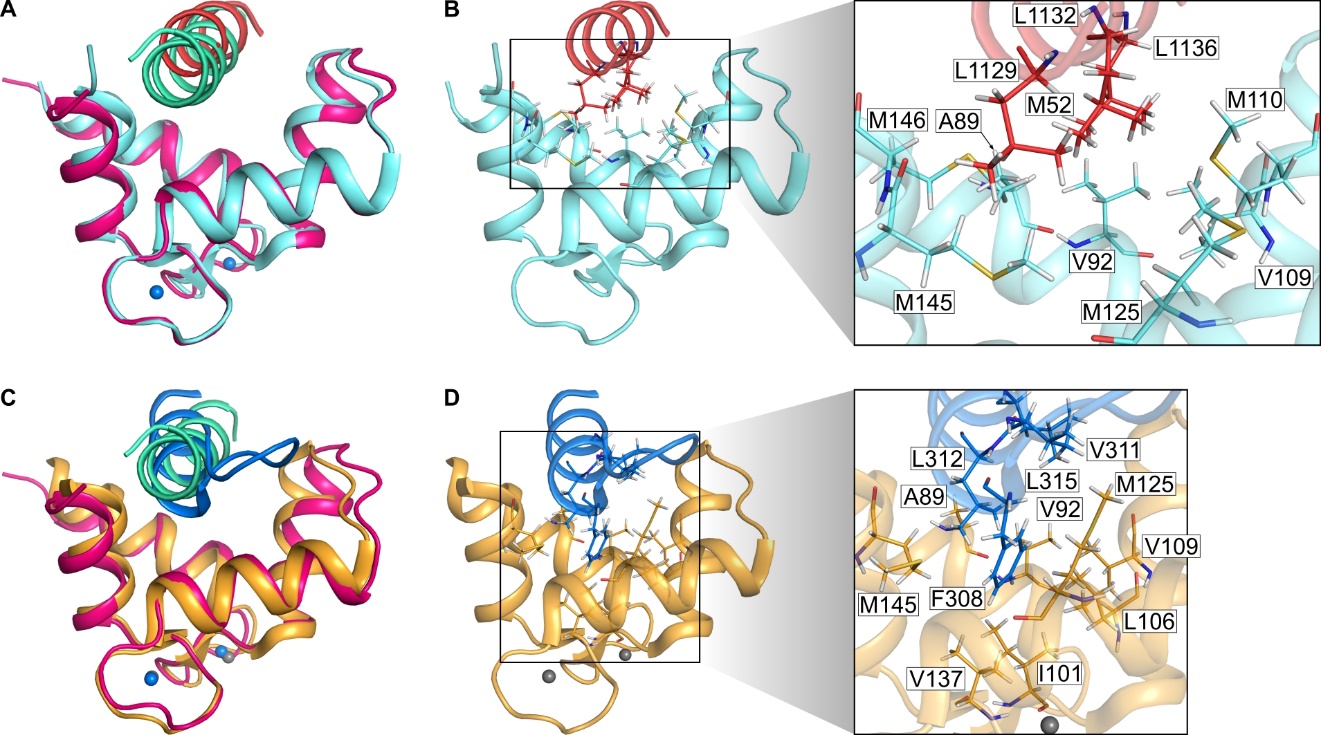


**Figure S4. Structural comparison of Ca^2+^-CaM C-lobe bound to various target peptides. (A)** NMR-derived structures of Ca^2+^-CaM C-lobe (magenta) bound to GluN2A-C0 peptide (green) overlaid onto Ca^2+^-CaM C-lobe (cyan) bound to CNGB1 CaM2 peptide (in red, PDB ID: 8DGH (5)). **(B)** Ribbon representation of Ca^2+^-CaM C-lobe bound to the CNGB1 CaM2 peptide. Expanded view of the boxed region shows residues at the binding interface highlighted as sticks and labeled. **(C)** NMR-derived structure of Ca^2+^-CaM C-lobe (magenta) bound to GluN2A-C0 peptide (green) overlaid onto the x-ray structure of Ca^2+^-CaM C-lobe (orange) bound to the creatine kinase (CK) peptide (blue, PDB ID: 7BF1 (6)). **(D)** Ribbon representation of Ca^2+^-CaM C-lobe bound to the CK peptide. Expanded view of residues at the binding interface highlighted as sticks and labeled. Bound Ca^2+^ ions are represented as gray spheres. Ca^2+^ ions are represented in blue (for Ca^2+^-CaM C-lobe bound to GluN2A peptide) or gray (for Ca^2+^-CaM C-lobe bound to CNGB1 CaM2 and CK peptides).

**Figure S5. Structural model of the NMDAR tetramer bound to 4 molecules of Ca^2+^-CaM generated by AlphaFold3.** **(A)** Ribbon diagram of the AlphaFold3 modeled NMDAR tetramer bound to 4 Ca^2+^-CaM. GluN1, GluN2A, and bound CaM are colored red, blue, and cyan (magenta), respectively. Four molecules of CaM (CaM1, CaM2, CaM3, and CaM4) are bound to the NMDAR tetramer. CaM1 and CaM2 are colored cyan and CaM3 and CaM4 are colored magenta. **(B)** Expanded view of four CaM bound to C0 helices of GluN1 (red) and GluN2A (blue). The CaM1 C-lobe (cyan) is bound to GluN1 C0 helix (red) and the CaM3 C-lobe (magenta) is bound to GluN2A C0 helix (blue). The C-lobe atoms of CaM1 and CaM3 (or CaM2 and CaM4) are spatially overlapped in the AlphaFold3 model, which is energetically not valid. CaM1 (cyan) and CaM3 (magenta) are related by symmetry to CaM2 (cyan) and CaM4 (magenta).

**SI References**

1. Abramson, J., Adler, J., Dunger, J., Evans, R., Green, T., Pritzel, A., Ronneberger, O., Willmore, L., Ballard, A. J., Bambrick, J., Bodenstein, S. W., Evans, D. A., Hung, C.-C., O’Neill, M., Reiman, D., Tunyasuvunakool, K., Wu, Z., Žemgulytė, A., Arvaniti, E., Beattie, C., Bertolli, O., Bridgland, A., Cherepanov, A., Congreve, M., Cowen-Rivers, A. I., Cowie, A., Figurnov, M., Fuchs, F. B., Gladman, H., Jain, R., Khan, Y. A., Low, C. M. R., Perlin, K., Potapenko, A., Savy, P., Singh, S., Stecula, A., Thillaisundaram, A., Tong, C., Yakneen, S., Zhong, E. D., Zielinski, M., Žídek, A., Bapst, V., Kohli, P., Jaderberg, M., Hassabis, D., and Jumper, J. M. (2024) Accurate structure prediction of biomolecular interactions with AlphaFold 3. *Nature*. **630**, 493–500

2. Cudia, D. L., Ahoulou, E. O., Bej, A., Janssen, A. N., Scholten, A., Koch, K.-W., and Ames, J. B. (2024) NMR Structure of Retinal Guanylate Cyclase Activating Protein 5 (GCAP5) with R22A Mutation That Abolishes Dimerization and Enhances Cyclase Activation. *Biochemistry*. **63**, 1246–1256

3. Fallon, J. L., Halling, D. B., Hamilton, S. L., and Quiocho, F. A. (2005) Structure of Calmodulin Bound to the Hydrophobic IQ Domain of the Cardiac Cav1.2 Calcium Channel. *Structure*. **13**, 1881–1886

4. Irene, D., Huang, J.-W., Chung, T.-Y., Li, F.-Y., Tzen, J. T.-C., Lin, T.-H., and Chyan, C.-L. (2013) Binding orientation and specificity of calmodulin to rat olfactory cyclic nucleotide-gated ion channel. *J. Biomol. Struct. Dyn.* **31**, 414–425

5. Bej, A., and Ames, J. B. (2022) NMR Structures of Calmodulin Bound to Two Separate Regulatory Sites in the Retinal Cyclic Nucleotide-Gated Channel. *Biochemistry*. **61**, 1955–1965

6. Sprenger, J., Trifan, A., Patel, N., Vanderbeck, A., Bredfelt, J., Tajkhorshid, E., Rowlett, R., Lo Leggio, L., Åkerfeldt, K. S., and Linse, S. (2021) Calmodulin complexes with brain and muscle creatine kinase peptides. *Curr. Res. Struct. Biol.* **3**, 121–132
